# Role of DNA methylation in the relationship between glioma risk factors and glioma incidence: a two-step Mendelian randomization study

**DOI:** 10.1038/s41598-023-33621-1

**Published:** 2023-04-21

**Authors:** Amy E. Howell, Caroline Relton, Richard M. Martin, Jie Zheng, Kathreena M. Kurian

**Affiliations:** 1 Brain Tumour Research Centre, Institute of Clinical Neurosciences, University of Bristol, Bristol, UK; 2 MRC Integrative Epidemiology Unit (IEU), Bristol Medical School, University of Bristol, Oakfield House, Oakfield Grove, Bristol, BS8 2BN UK; 3 National Institute for Health Research (NIHR) Bristol Biomedical Research Centre, University Hospitals Bristol and Weston NHS Foundation Trust and University of Bristol, Bristol, UK; 4 Department of Endocrine and Metabolic Diseases, Shanghai Institute of Endocrine and Metabolic Diseases, Ruijin Hospital, Shanghai Jiao Tong University School of Medicine, Shanghai, China; 5 Shanghai National Clinical Research Center for Metabolic Diseases, Key Laboratory for Endocrine and Metabolic Diseases of the National Health Commission of the PR China, Shanghai National Center for Translational Medicine, Ruijin Hospital, Shanghai Jiao Tong University School of Medicine, Shanghai, China

**Keywords:** Diseases, Health care, Neurology, Risk factors

## Abstract

Genetic evidence suggests glioma risk is altered by leukocyte telomere length, allergic disease (asthma, hay fever or eczema), alcohol consumption, childhood obesity, low-density lipoprotein cholesterol (LDLc) and triglyceride levels. DNA methylation (DNAm) variation influences many of these glioma-related traits and is an established feature of glioma. Yet the causal relationship between DNAm variation with both glioma incidence and glioma risk factors is unknown. We applied a two-step Mendelian randomization (MR) approach and several sensitivity analyses (including colocalization and Steiger filtering) to assess the association of DNAm with glioma risk factors and glioma incidence. We used data from a recently published catalogue of germline genetic variants robustly associated with DNAm variation in blood (32,851 participants) and data from a genome-wide association study of glioma risk (12,488 cases and 18,169 controls, sub-divided into 6191 glioblastoma cases and 6305 non-glioblastoma cases). MR evidence indicated that DNAm at 3 CpG sites (cg01561092, cg05926943, cg01584448) in one genomic region (*HEATR3*) had a putative association with glioma and glioblastoma risk (False discovery rate [FDR] < 0.05). Steiger filtering provided evidence against reverse causation. Colocalization presented evidence against genetic confounding and suggested that differential DNAm at the 3 CpG sites and glioma were driven by the same genetic variant. MR provided little evidence to suggest that DNAm acts as a mediator on the causal pathway between risk factors previously examined and glioma onset. To our knowledge, this is the first study to use MR to appraise the causal link of DNAm with glioma risk factors and glioma onset. Subsequent analyses are required to improve the robustness of our results and rule out horizontal pleiotropy.

## Introduction

Brain tumours such as glioma are responsible for the greatest number of years lost to cancer to those under 40 years of age^1^ despite having age adjusted incidence rates ranging from just 4.67 to 5.73 per 100,000^2,3^. A serious health burden is posed by glioma due to their poor prognosis, with an overall 5-year survival rate of under 20% and significant morbidity in survivors^4–6^. While there have been many attempts to ascertain risk factors for glioma, evidence has been inconsistent, and the aetiology of glioma remains largely unclear^7–23^.

Mendelian randomization (MR) studies have provided some evidence to implicate genetically predicted leukocyte telomere length, allergic diseases (asthma, hay fever or eczema), alcohol consumption, childhood extreme obesity, low-density lipoprotein cholesterol (LDLc) and triglyceride levels as causally relevant risk factors for glioma^24^. The underlying biological mechanisms by which these traits causally relate to glioma risk remains to be established.

One approach to understanding the aetiological pathways influencing glioma onset is to exploit the increasing body of molecular phenotype data to examine epigenetic pathways. Epigenetic changes include chemical modifications that do not change the sequence of DNA but can alter gene expression^25^. The most commonly measured form of epigenetic mark is DNA methylation (DNAm), whereby a methyl group (–CH_3_) is either added or subtracted to a cytosine nucleotide adjacent to a guanine nucleotide within the DNA sequence (cytosine-phosphate-guanine [CpG] site)^25^. One method to examine DNAm variation linked to glioma incidence is to undertake an epigenome-wide association study (EWAS)^26–36^. However, most EWAS have been limited by very modest sample sizes or have been undertaken using glioma tumour tissue which are potentially biased through confounding by treatment thus restricting any inferences that can be made with respect to disease aetiology.

**Figure 1:**
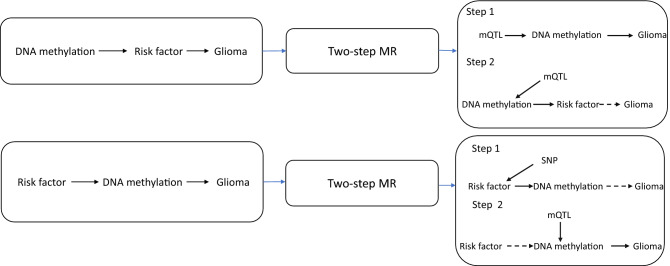
The research questions and how they link to causal pathways in glioma development. An overview displaying the objective of each analysis, the techniques and causal mechanisms examined. *DNAm* DNA methylation, *SNP* single nucleotide polymorphism, *mQTL* methylation quantitative trait loci.

As recent studies have reported that DNAm influences glioma-related traits including allergic diseases^37^, telomere length^38^ childhood obesity^39^ and glioma risk^40^, we sought to assess the causal relationship of DNAm with glioma risk factors identified in a prior study^24^ (Table 1) and glioma incidence using two-step MR^41^. We used a recently published catalogue of germline genetic variants robustly associated with DNAm variation in blood, namely methylation quantitative trait loci (mQTL)^42^, as a proxy for DNAm variation in blood, rather than measuring DNAm variation directly. As glioma is a disease with a high degree of heterogeneity, with differing genetic profiles both intra- and inter-tumourally^43^, we performed a subtype analysis by splitting the glioma outcome data into glioblastoma or non-glioblastoma. An overview of the research questions can be found in Fig. 1.

**Table 1:** Summary of the risk factors identified in a previous study and their effect on glioma or glioma subtype.

Risk factor	Outcome	OR (95% CI)	*P*-value
Alcohol consumption	Glioma	4.42 (1.07–18.32)	4.05 × 10^–02^
Alcohol consumption	Glioblastoma	8.37 (1.69–41.54)	9.36 × 10^–03^
Allergic disease	Glioblastoma	1.29 (1.01–1.67)	4.76 × 10^–02^
low-density lipoprotein cholesterol	Non-glioblastoma	0.79 (0.63–0.99)	3.99 × 10^–02^
Obesity (childhood extreme)	Glioma	1.11 (1.02–1.21)	1.63 × 10^–02^
Obesity (childhood extreme)	Glioblastoma	1.12 (1.02–1.22)	2.07 × 10^–02^
Telomere length	Glioma	4.09 (1.13–14.86)	3.24 × 10^–02^
Telomere length	Non-glioblastoma	4.05 (1.72–9.56)	1.38 × 10^–03^
Triglycerides	Non-glioblastoma	0.77 (0.59–1.00)	4.86 × 10^–02^

*OR* change in glioma risk per standard deviation change in risk factor, *95% CI* 95% confidence intervals, *p-value* p-value for the observed effect.

## Results

### Does DNAm causally influence both glioma risk and glioma risk factors?

Using the full summary statistics for the 232,476 CpG sites (n = 32,851) reported in GoDMC, instrumental variables (IVs) were constructed (*P* < 5 × 10^−8^ and *r*^2^ < 0.001) to act as a proxy for 42,659 CpG sites that could be used in a two-sample MR framework.

Two-sample MR was used to investigate the potential causal effect of DNAm variation at 42,659 CpG sites and glioma risk. For glioma risk there was MR evidence for 284 CpG-glioma effects that met the false discovery rate (FDR) correction threshold (< 0.05). MR results that met the FDR threshold can be found in Appendix 1.1. F-statistic calculations indicated that all 284 CpG sites linked to glioma had an F-statistic > 10 (Appendix 1.2) which suggests that the MR estimate was less likely to be affected by weak instrument bias.

**Figure 2:**
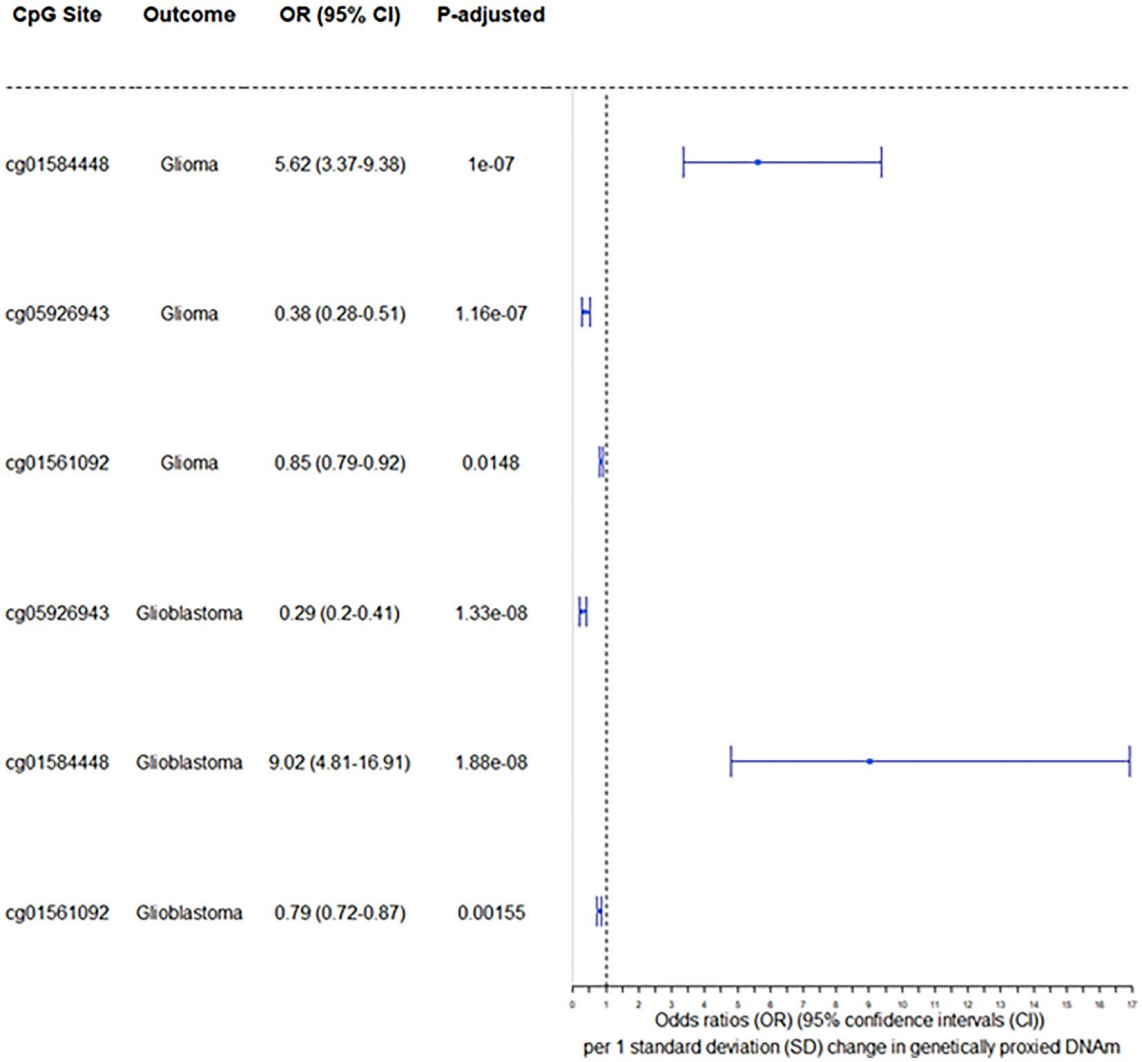
CpG sites that showed robust evidence of a causal role on glioma risk. Forest plot of CpG sites that showed robust MR evidence of an association with glioma or glioblastoma and colocalized with glioma or glioblastoma. OR, per standard deviation change in genetically proxied DNA methylation; 95% CI, 95% confidence intervals; p-adjusted, p-value adjusted for FDR for the observed effect.

As a sensitivity analysis, colocalization was used to establish the probability that DNAm and glioma were driven by the same causal variant at each locus. In the colocalization analyses, we found suggestive evidence (H_4_ > 70%) that DNAm at 3 of the 284 CpG sites and glioma were driven by the same genetic variant. Next, we examined the directionality of DNAm at the 3 CpG sites and glioma risk using the Steiger filtering method: the 3 CpG sites showed evidence that the direction of effect was methylation influencing glioma risk (Fig. 2). Complete results from both MR and sensitivity analysis are summarised in Table 2.

**Table 2:** CpG sites that met the FDR correction threshold (p-value < 0.05) in the MR analyses of glioma risk, showed evidence of colocalization (H_4_ > 0.7) and the correct direction of effect.

Outcome	CpG site	Number of SNPs	OR (95% CI)	*p*-adjusted	H_4_ > 0.8	H_4_ > 0.7	Steiger direction
Glioma	cg01584448	1	5.62 (3.37–9.38)	1.00E−07	FALSE	TRUE	TRUE
Glioma	cg05926943	1	0.38 (0.28–0.51)	1.16E−07	FALSE	TRUE	TRUE
Glioma	cg01561092	2	0.85 (0.79–0.92)	1.48E−02	FALSE	TRUE	TRUE

*OR* odds ratio per standard deviation change in methylation, *95% CI* 95% confidence intervals, *p-value* p-value for the observed effect, *SNP* single nucleotide polymorphism.

In the subtype analysis, there were 209 CpG-glioblastoma (F-statistic > 10) MR estimates that met the FDR correction threshold (FDR < 0.05) (Appendix 1.3). 3 CpG-glioblastoma associations showed evidence of colocalization and all 3 CpG sites showed evidence that the direction of effect was methylation influencing glioblastoma risk (Fig. 2). The full MR results and results from each sensitivity analysis is summarised in Table 3.

**Table 3:** CpG sites that met the FDR correction threshold < 0.05 in the MR analyses against glioblastoma, showed evidence of colocalization (H_4_ > 0.7) and the correct direction of effect.

Outcome	Exposure	Number of SNPs	OR (95% CI)	*p*-adjusted	H_4_ > 0.8	H_4_ > 0.7	Steiger direction
Glioblastoma	cg05926943	1	0.29 (0.20–0.41)	1.33E−08	FALSE	TRUE	TRUE
Glioblastoma	cg01584448	1	9.02 (4.81–16.91)	1.88E−08	FALSE	TRUE	TRUE
Glioblastoma	cg01561092	2	0.79 (0.72–0.87)	1.55E−03	FALSE	TRUE	TRUE

*OR* odds ratio per standard deviation change in methylation, *95% CI* 95% confidence intervals, *p-value* p-value for the observed effect, *SNP* single nucleotide polymorphism.

For the glioma subtypes there were 175 CpG-non-glioblastoma effects (F-statistic > 10) that met the FDR correction threshold (< 0.05) (Appendix 1.4). Of these 175 CpG sites, 0 CpG-non-glioblastoma effects showed strong evidence of colocalization.

**Figure 3:**
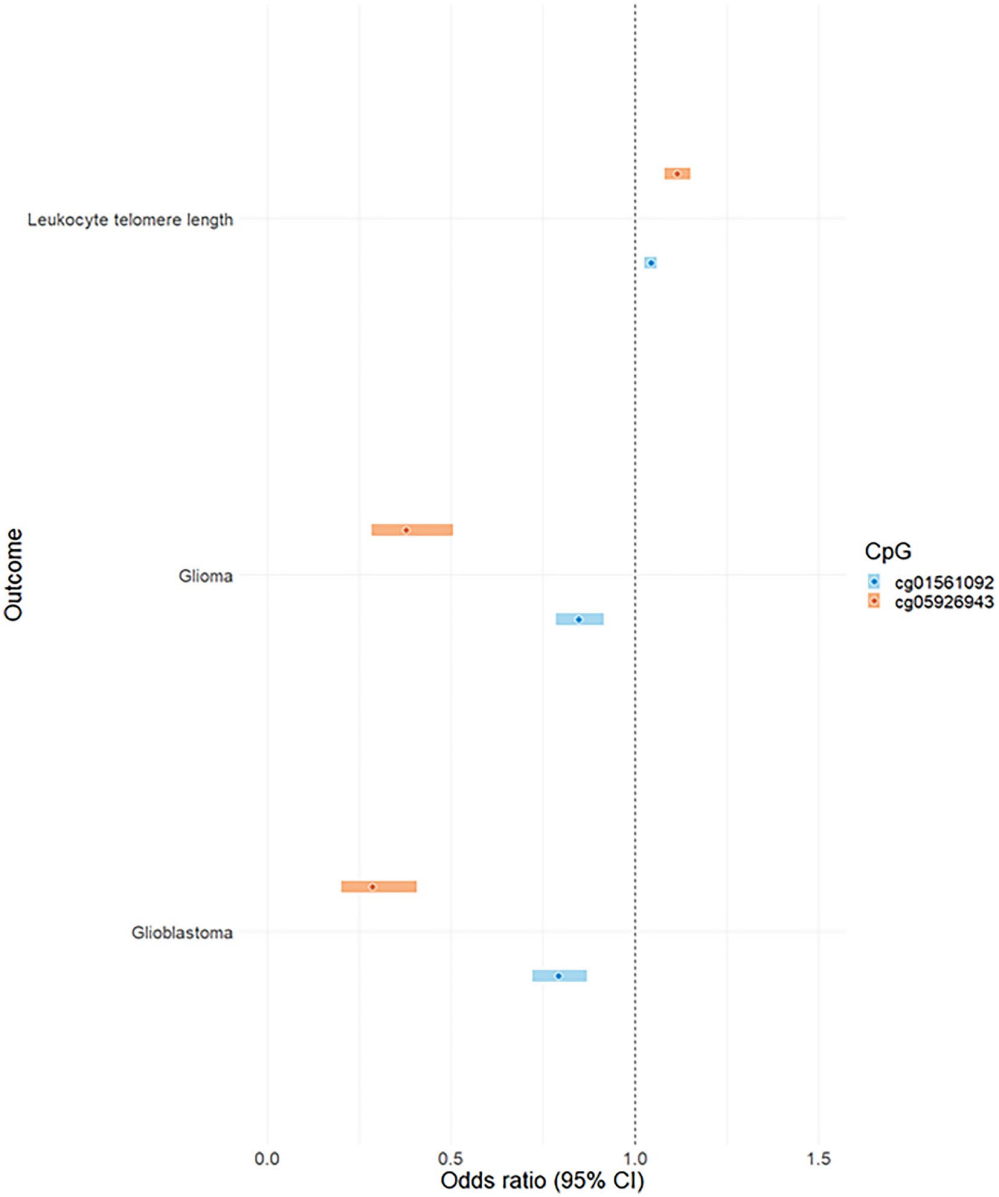
The MR estimates of CpG methylation on glioma, glioblastoma and telomere length. The association between that associated with glioma, glioblastoma and telomere length; OR (95%) is the effect of DNAm on glioma, glioblastoma and telomere length. MR effect estimates are reported as odds ratios (95% confidence intervals (CI)) per 1 standard deviation change in genetically proxied DNAm.

The 3 CpG sites that showed MR and colocalization evidence of an association with glioma and glioblastoma are displayed in Fig. 3. In summary, the results indicate that increased levels of DNAm at cg01584448 increases risk of glioma (OR 5.62, 95% CI 3.37–9.36, *p*-adjusted 1 × 10^− 7^) and glioblastoma (OR 9.02, 95% CI 4.81–16.91, *p*-adjusted 1.88 × 10^− 8^). cg5926943 and cg01561092 were associated with a decrease in the risk of both glioma (OR 0.38, 95% CI 0.28–0.51, *p*-adjusted 1.16 × 10^− 7^; OR 0.85, 95% CI 0.79–0.92, *p*-adjusted 1.48 × 10^− 2^) and glioblastoma (OR 0.29, 95% CI 0.20–0.41, *p*-adjusted 1.33 × 10^− 8^; OR 0.79, 95% CI 0.72–0.87, *p*-adjusted 1.55 × 10^− 3^), respectively.

#### Appraising the causal role of DNA methylation on glioma risk factors

We performed two-sample MR to examine the causal role of DNAm variation at the 3 CpG sites altering risk of glioma or glioma subtypes with glioma risk factors. The results from the extensive analysis are present in Table 4. We identified 5 associations that survived the FDR corrected *p*-value threshold (*p*-adjusted < 0.05). Two of these associations were robust to colocalization and Steiger filtering. The results indicate that DNAm variation at cg05926943 and cg01561092 are associated with an increase in telomere length (OR 1.12, 95% CI 1.08–1.15, *p*-adjusted 3.90 × 10^− 11^: OR 1.04, 95% CI 1.03–1.06, *p*-adjusted 8.96 × 10^− 7^), respectively (Fig. 3).

**Table 4:** The Mendelian randomization, colocalization and Steiger filtering results for the MR analysis of DNAm on glioma related traits.

Exposure	Outcome	OR (95% CI)	*P*-value	*P*-value adjusted	*P*-value < 0.05	Steiger direction	H_4_ > 0.8
cg01561092	Alcohol consumption	1.02 (0.99–1.04)	0.170	0.340	FALSE	–	–
cg01584448	Alcohol consumption	1.01 (0.93–1.09)	0.868	1.16	FALSE	–	–
cg05926943	Alcohol consumption	1.00 (0.96–1.04)	0.987	1.05	FALSE	–	–
cg01561092	Allergic disease (asthma, hay fever or eczema)	0.99 (0.94–1.04)	0.628	1.00	FALSE	–	–
cg01584448	Allergic disease (asthma, hay fever or eczema)	1.01 (0.86–1.18)	0.949	1.17	FALSE	–	–
cg05926943	Allergic disease (asthma, hay fever or eczema)	1.00 (0.91–1.09)	0.987	0.987	FALSE	–	–
cg01584448	Childhood obesity	0.81 (0.39–1.68)	0.565	1.00	FALSE	–	–
cg01584448	LDL cholesterol	0.94 (0.88–0.99)	0.0248	0.0566	FALSE	–	–
cg05926943	LDL cholesterol	1.04 (1.00–1.07)	0.0243	0.0648	FALSE	–	–
cg01561092	LDL cholesterol	1.00 (0.98–1.02)	0.950	1.086	FALSE	–	–
cg01584448	Telomere length	0.82 (0.77–0.86)	8.88E−13	1.421E-11	TRUE	TRUE	FALSE
cg05926943	Telomere length	1.12 (1.08–1.15)	4.87E−12	3.896E-11	TRUE	TRUE	TRUE
cg01561092	Telomere length	1.04 (1.03–1.06)	1.68E−07	8.96E-07	TRUE	TRUE	TRUE
cg01584448	Triglycerides	0.92 (0.87–0.97)	0.00260	0.00831	TRUE	TRUE	FALSE
cg05926943	Triglycerides	1.05 (1.02–1.08)	0.00240	0.00961	TRUE	TRUE	FALSE
cg01561092	Triglycerides	1.00 (0.98–1.01)	0.725	1.05	FALSE	–	–

*OR* odds ratio (95% confidence intervals [CI]) per 1 standard deviation change in genetically proxied DNA methylation.

#### Overlap with gene expression

DNAm variation at the 3 CpG sites (cg01561092, cg05926943, cg01584448) found to putatively influence glioma and glioblastoma risk were used to investigate hypothesis driven tissue-specific effects. We hypothesised that DNAm that influences glioma and glioblastoma risk may be influenced by gene expression in blood and brain tissue. All 3 CpG sites were annotated to the gene *HEATR3* (Ensemble ID ENSG00000155393).

To evaluate the association of gene expression with glioma and glioblastoma risk at *HEATR3* in blood tissue, instruments were constructed using eQTLGen Consortium (n = 31,684).

In the MR analysis, we observed evidence that survived the FDR corrected *p*-value threshold (*p*-adjusted < 0.05), colocalization and Steiger filtering, that gene expression at *HEATR3* was associated with an increase in glioma risk (OR 1.20, 95% CI 1.11–1.29, *p*-adjusted 7.61 × 10^− 6^) and an increase in glioblastoma risk (OR 1.28, 95% CI 1.16–1.41, *p*-adjusted 2.54 × 10^− 7^) (Table 5).

**Table 5:** The MR results for the analysis of differential gene expression in blood tissue with glioma and glioblastoma risk.

Outcome	Exposure	*p*-adjusted	OR (95% CI)	H_4_ > 0.8	Steiger direction
Glioma	*HEATR3*	7.61E−06	1.20 (1.11–1.29)	TRUE	TRUE
Glioblastoma	*HEATR3*	2.54E−07	1.28 (1.16–1.41)	TRUE	TRUE

P-adjusted, p-value adjusted for FDR. MR effect estimates are reported as odds ratios (95% confidence intervals (CI)) per 1 standard deviation change in genetically proxied differential gene expression. SNP, single nucleotide polymorphism.

**Figure 4:**
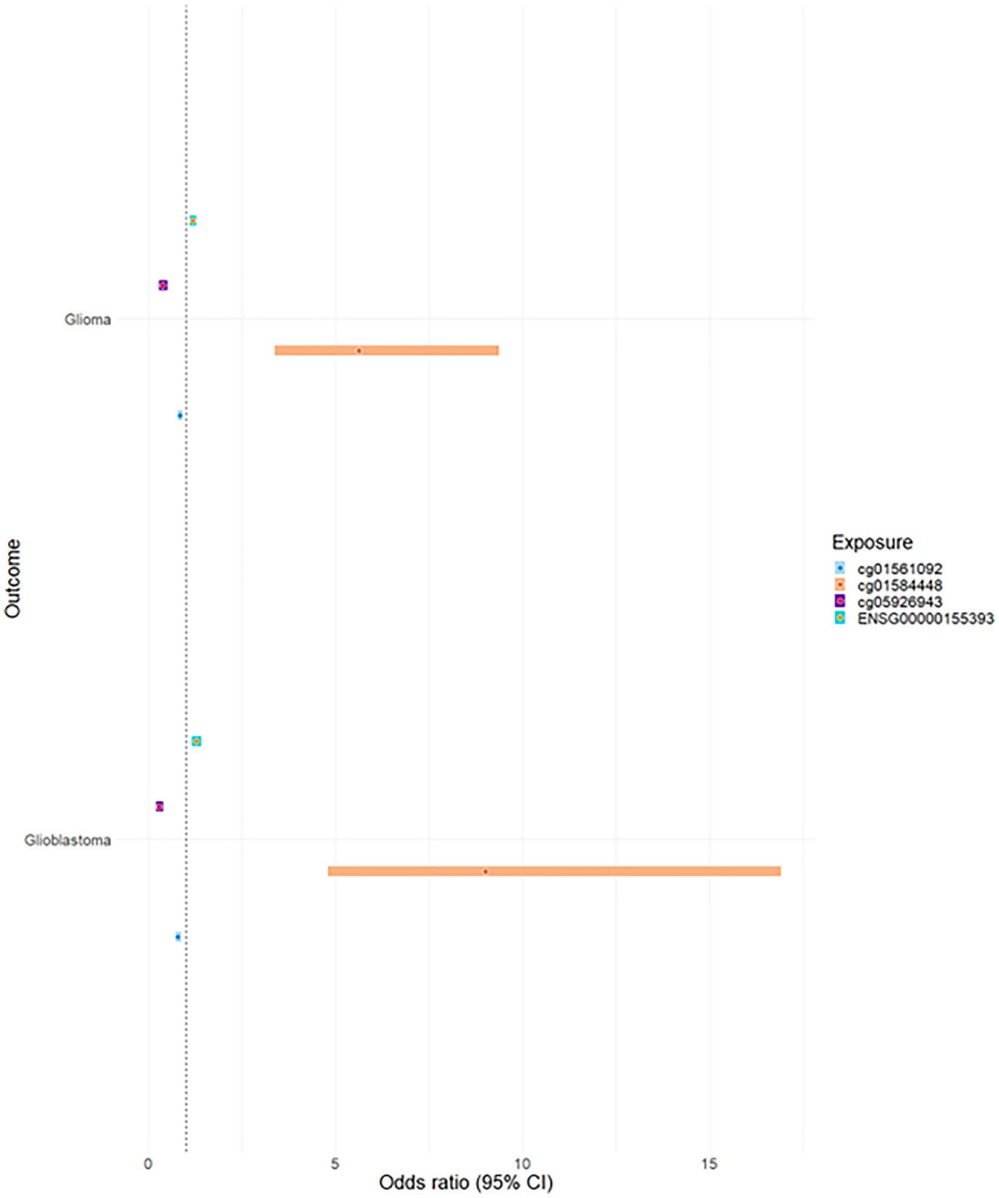
A comparison of MR estimates. Comparison between DNA methylation (DNAm) and gene expression. MR effect estimates are reported as odds ratios (95% confidence intervals (CI)) per 1 standard deviation change in genetically proxied DNAm.

When comparing the DNAm MR results with the gene expression MR results, the direction of effect estimated for *HEATR3* is consistent with cg01584448. The direction of the estimated effect for the two CpG sites (cg01561092, cg05926943) was discordant with gene expression (Fig. 4).

To establish if the associations between the CpG sites and glioma is mediated by changes in gene expression at *HEATR3* in blood tissue we applied “moloc”. Moloc assessed the likelihood that DNAm, gene expression and glioma susceptibly are driven by the same causal variant. The results indicated suggestive evidence (PPA > 70%) of colocalization between gene expression and glioma (but not by DNAm at cg01561092). Similarly, colocalization between DNAm and glioma at cg05926943 was observed but not with gene expression. The results provided evidence of two distinct causal variants for methylation and expression at cg01584448 (Table 6).

**Table 6:** The results from the moloc analysis.

(eqtl = E, mqtl = DM, trait = G)																	
Trait	Tag SNP	CpG	E	E,DM	E,G	E,DMG	E,DM,G	DM	DM,G	EG.DM	G	EDM,G	EDM	EG	DMG	EDMG	NULL
Glioma	rs2356838	cg01561092	0	1.60E−07	0	0.19	0.08	0	0	**0.72**	0	5.47E−22	1.06E−27	0	0	1.18E−21	0
Glioma	rs4238851	cg01584448	0	**0.97**	0	0.00	0.02	0	0	0.01	0	0	0	0	0	0	0
Glioma	rs8047504	cg05926943	0	1.99E−07	0	**0.78**	0.10	0	0	0.12	0	0	0	0	0	0	0
Glioblastoma	rs2287197	cg01561092	0	2.63E−08	0	0.67	0.07	0	0	0.26	0	0	0	0	0	0	0
Glioblastoma	rs12102426	cg01584448	0	**9.77E−01**	0	0.00	0.02	0	0	0.00	0	0	0	0	0	0	0
Glioblastoma	rs1547478	cg05926943	0	1.46E−08	0	0.33	0.04	0	0	0.63	0	0	0	0	0	0	0

Significant values are in bold.

The columns provide the posterior probability (PPA) for each colocalization scenario where E = eQTL, DM = mQTL, G = trait. The trait is provided in the first column. A PPA > 0.7 was used as suggestive evidence for that scenario and PPA > 0.8 was used as strong evidence.

Next, to establish if there was an association between gene expression and glioma or glioblastoma risk at *HEATR3* in brain tissue, instruments were constructed using data from GTEx v8 (n = 1194).

The two associations from the MR analysis survived the FDR corrected *p*-value threshold, however, neither showed evidence of colocalization suggesting the MR result may be biased by genetic confounding. The results from the extensive analyses are provided Table 7.

**Table 7:** The Mendelian randomization results for the analysis of differential gene expression in brain with glioma and glioblastoma risk.

Outcome	*P*-value	OR (95% CI)	H_4_ > 0.7
Glioma	4.62E−10	1.12 (1.08–1.16)	FALSE
Glioblastoma	8.87E−11	1.15 (1.11–1.21)	FALSE

P-adjusted, p-value adjusted for FDR. MR effect estimates are reported as odds ratios (OR) (95% confidence intervals (CI)) per 1 standard deviation change in differential gene expression.

### Does DNA methylation mediate the effect of risk factors on glioma?

#### Appraising the causal role of glioma risk factors on DNAm

We performed two-sample MR to investigate the potential causal role of allergic disease, triglycerides, LDLc, alcohol consumption, telomere length and childhood obesity with DNAm variation at 42,659 CpG sites. The MR analysis indicated little evidence of a causal role for any of the glioma related traits on DNAm variation (Bonferroni corrected *P* value < 0.0083) (Table 8).

**Table 8:** The MR effect estimates of the effect of the glioma related trait on CpG methylation.

Glioma related traits	Units of trait	Number of SNPs used an IV	Beta (SD increase in DNAm per unit increase in the trait)	95% CI	*p*-value
Telomere length	Kilobases SD = 0.65	3	− 2.33	(− 5.98–1.31)	0.211
Allergic disease (asthma, hay fever or eczema)	LogOR	66	0.38	(− 0.76–1.52)	0.514
Childhood obesity	LogOR	226	− 0.22	(− 2.67–2.23)	0.859
Alcohol consumption	SD = one additional drink per week	33	3.69	(− 3.86–11.24)	0.339
LDL cholesterol	SD = 3.57 mmol/L	136	0.34	(− 0.99–1.67)	0.621
Triglycerides	SD = 1.50 mmol/L	251	− 0.44	(− 1.50–0.62)	0.411

*SD* standard deviation, *95% CI* confidence intervals; p-value for the observed effect.

## Discussion

Extensive analyses were conducted to establish the role of DNAm on the causal pathway leading to glioma onset. MR evidence robust to the FDR *p*-value threshold and Steiger filtering identified 3 CpG sites (cg01561092, cg05926943, cg01584448) in one genomic region (*HEATR3*) that have a putative association with glioma and glioblastoma risk. In support of these findings, MR provided evidence that higher levels of gene expression of *HEATR3* in blood tissue was associated with an increased risk of glioma and glioblastoma. MR provided little evidence to suggest any CpG sites influenced non-glioblastoma. By examining the role of DNAm variation at these 3 CpG sites with putative glioma related traits (alcohol consumption, allergic disease, childhood obesity, LDL cholesterol, triglycerides, and telomere length), we report evidence that 2 of these CpG sites (cg01561092, cg05926943) influenced telomere length. MR offered little evidence to suggest that DNAm acts as a mediator on the causal pathway between glioma related traits previously examined and glioma onset.

Higher levels of methylation at cg01584448 were associated with an increase in glioma and glioblastoma risk. Whereas higher levels of methylation at cg5926943 and cg01561092 were associated with a lower risk of glioma and glioblastoma. To elucidate the observed putative association, the CpG sites were annotated to their closest gene. As the CpG sites reside in close genomic positions they were mapped to the same gene, a known oncogene, *HEATR3*, which has been associated with glioma risk in previous studies^44–46^; thus, providing evidence that the genomic region is relevant. Here MR, colocalization and Steiger filtering offered further evidence that differential gene expression of *HEATR3* within blood tissue increased the risk of glioma and glioblastoma. A conflicting pattern of DNAm was observed for cg5926943 and cg01561092 as they displayed an opposite correlation with gene expression. A prior study reported an inverse correlation between DNAm and gene expression for various CpGs and their closest gene, in several cancers^47^. Similarly, Houshdaran et al. reported that DNAm inversely correlated with gene expression in ovarian cancer cell lines^48^. Thus, it is possible that the inverse correlation indicates co-regulation of DNAm and gene expression with glioma development.

Due to the complex nature of this interaction between DNAm and gene expression, moloc was implemented to establish if glioma, DNAm and gene expression shared a common causal genetic variant, to provide further supporting evidence of an underlying causal association between these traits rather than findings being driven through genetic confounding (e.g., LD between an mQTL and a variant influencing glioma risk). The results from the moloc analysis indicated that gene expression colocalizes with glioma but not with DNAm at cg01561092. Similarly, colocalization between DNAm and glioma at cg05926943 was observed but not with gene expression. There was evidence of two distinct causal variants for methylation and expression at cg01584448. There is evidence of colocalization between two of the traits at each CpG site (gene expression and glioma risk; methylation and glioma risk) thus it is possible that gene expression is under the control of methylation of a region rather than specific CpG sites.

The incidence and mortality of high-grade glioma increases with age, with the median age at diagnosis of 64 years^49^. The 3 CpG sites putatively associated with glioma risk in this study have been linked to age in previous EWAS^50^. Age-specific differences in glioma susceptibility could reveal clues about glioma aetiology. Additionally, previous models of age, based on DNAm have demonstrated an ability to predict the risk of both disease and survival in pre-cancerous tissue, including brain tissue^51–53^. These findings provide a rationale to evaluate whether an association exists between these epigenetic markers and age at diagnosis in glioma and subsequently whether DNAm can act as a prognostic marker.

Prior epidemiological studies have reported that longer leukocyte telomere length is linked to an increased risk of glioma^24,54^. Here, we provide evidence to further elucidate the molecular mechanism between telomere length, DNAm and glioma risk. Contrary to previous studies, we observed evidence that DNAm influencing the CpG sites (cg01561092, cg05926943) decreased glioma risk and increased leukocyte telomere length. The conflicting correlation could be a result of the complexity of the association underlying glioma development. A noteworthy concern is that since methylation was studied in blood tissue, which is unlikely to accurately proxy DNAm in the brain, the associations may be biased by confounding by tissue heterogeneity.

There was little evidence to suggest the glioma related traits influence cancer development through DNAm. These null results could reflect the fact that DNAm is not a causal mediator between these traits and glioma onset, or it could be a consequence of this MR study being underpowered since the variance explained by the IV for the trait was limited. In an attempt to reduce weak instrument bias, we obtained the summary data to proxy the glioma related traits from GWAS with a large sample size to improve the reliability of the causal estimates and we only used SNPs with an F statistic greater than 10.

An important consideration in the interpretation of this analysis is explained in detail by Min JL et al.^42^. The blood measured mQTL data utilised in this chapter, obtained from the GoDMC data set^42^, cannot be regarded as mediating the genetic association to the trait even when there is colocalization evidence of a shared genetic variants. Rather, when DNAm shows evidence of colocalizing with a complex trait, such as glioma and telomere length, then this is likely due to common cause. Therefore, despite CpG sites showing evidence of colocalization, it is possible that second instrumental variable assumption has been violated, as there could be a common cause for both DNAm and glioma risk. To establish if the CpG sites identified here are truly implicated in glioma onset more detailed analyses are required to triangulate evidence and to fully understand the mechanistic pathways.

Another limitation of this study is the fact that we used single-instrument MR to examine causal relationships and consequently was not properly able to appraise possible horizontal pleiotropic effects. We took measures to minimise this possibility: instruments were limited to *cis*-mQTLs as *trans*-mQTLs are more likely to have effects on methylation and glioma risk via distinct mechanisms; and colocalization techniques were implemented to test whether the putative causal variant is shared by the exposure (e.g., risk factor or DNAm) and the outcome (e.g., glioma or DNAm)^55–57^ thus increasing the probability that the two traits have a shared causal mechanism^55,58^.

Despite these limitations, this analysis has numerous strengths, including the use of two-sample MR to examine the causal role of DNAm in glioma risk by exploiting a vast epigenetic resource and the largest glioma GWAS. Thus, leading to increased statistical power and precision of effect estimates. Furthermore, to ensure IVs were valid, genetic instruments were constructed using a strict inclusion criteria and quality control steps were undertaken. For example, only *cis*-variants were included and instrument strength was checked. In addition, the orientation of the causal effect was inferred to reduce the likelihood of reverse causation.

## Methods

Reported results from all analyses are MR effect estimates that met either the false discovery rate (FDR) threshold (when DNAm or gene expression is the exposure) or the Bonferroni-corrected *p*-value threshold (glioma related traits is the exposure), showed evidence of colocalization^59^ to rule out genetic confounding, and displayed little evidence to suggest reverse causation through Steiger filtering (Fig. 2)^60^. All MR analyses were conducted using the “TwoSampleMR” package in R studio (version 4.1.0) using the computational facilities of the Advanced Computing Research Centre, University of Bristol (http://www.bristol.ac.uk/acrc/).

**Figure 5:**
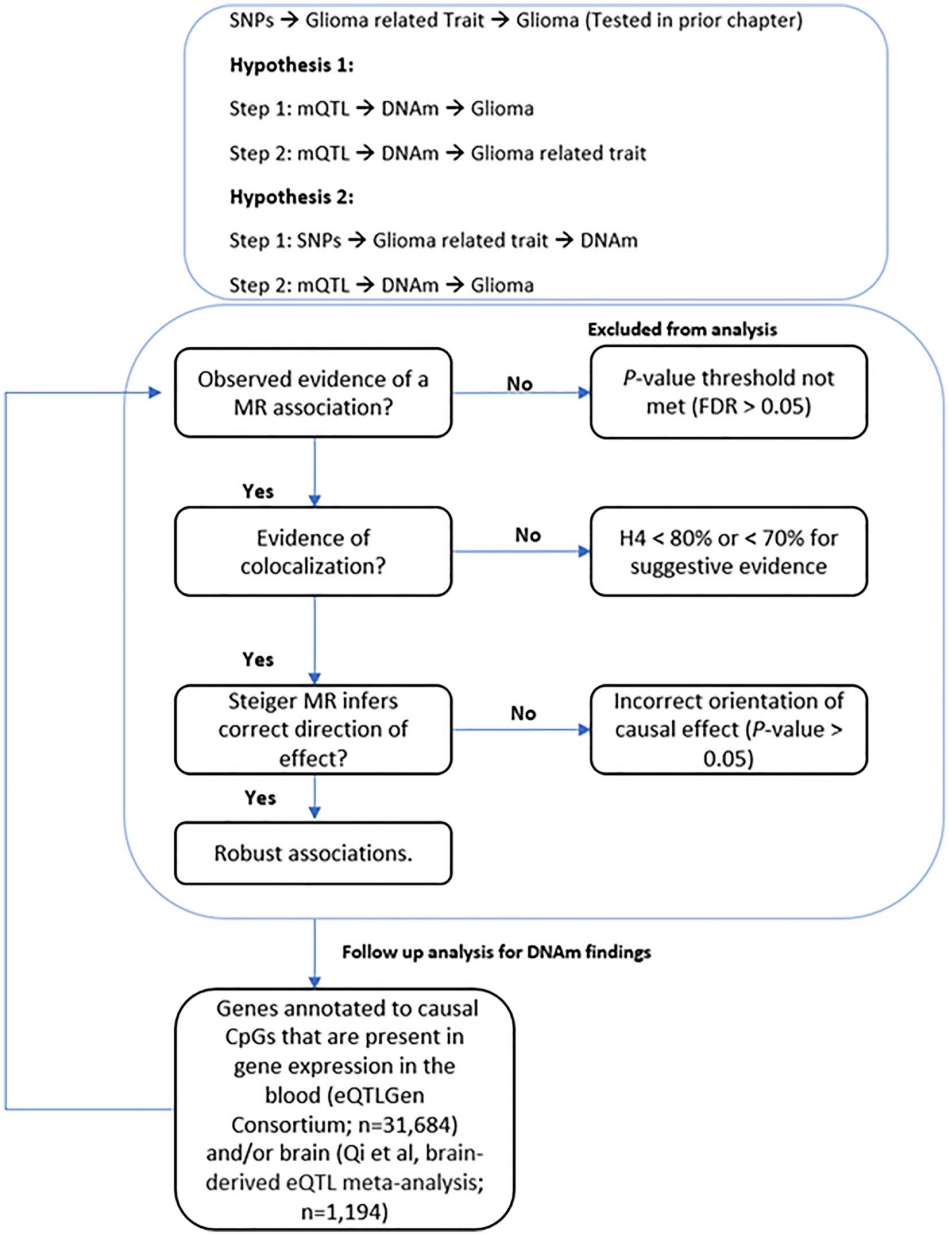
A summary of the MR pipeline. A summary of the analysis pipeline. All Mendelian randomization (MR) estimates were subject to further sensitivity analysis (colocalization and Steiger filtering) to enhance evidence for causal inference.

When DNAm or gene expression were instrumented as the exposure, we opted to use a more liberal FDR corrected *p*-value threshold, as we did not expect complete independence of all statistical tests (within overall glioma, glioblastoma, or non-glioblastoma analyses), compared to the Bonferroni *p*-value threshold used, when a glioma related trait was instrumented as the exposure.

### Mendelian randomization estimate

In cases where there was a single nucleotide polymorphism (SNP) to act as a proxy for the exposure of interest (e.g., DNAm), the causal effect estimates from MR were calculated using the Wald ratio (β_GD_/β_GP_)^61^ and standard errors approximated using the delta method^62^. Where the exposure (e.g., DNAm variation at a CpG site) was instrumented by multiple independent SNPs (r^2^ < 0.001), causal effect estimates were calculated using the random effects inverse variance weighted (IVW) method to allow overdispersion, where the Wald ratios were combined into a single causal estimate by meta-analysis^63^.

#### Colocalization

IV2 violations can occur through genetic confounding if genetic variants are correlated through linkage LD (Fig. 3). Therefore, for associations which met the *p*-value threshold (FDR < 0.05) we applied pairwise conditional and colocalization (PWCoCo)^57^ to determine whether the genetic variant associated with the exposure, e.g., DNAm, was the same genetic variant altering the outcome e.g., glioma (i.e., as identified in glioma genome wide association study [GWAS]), thus permitting evaluation of the presence of genetic confounding^64^. Colocalization requires providing prior probabilities that any random SNP within the genomic region of interest is associated with the exposure, the outcome or both (p1 = 1e−4, p2 = 1e−4, p12 = 1e−5). SNPs from a ± 250KBP window were extracted around the instrumented SNP(s) for each putative causal SNP from the exposure and outcome GWAS. A posterior probability for H_4_ > 0.8 was designated as “strong” and 0.7 > a posterior probability for H_4_ < 0.8 as “suggestive” evidence.

**Figure 6:**
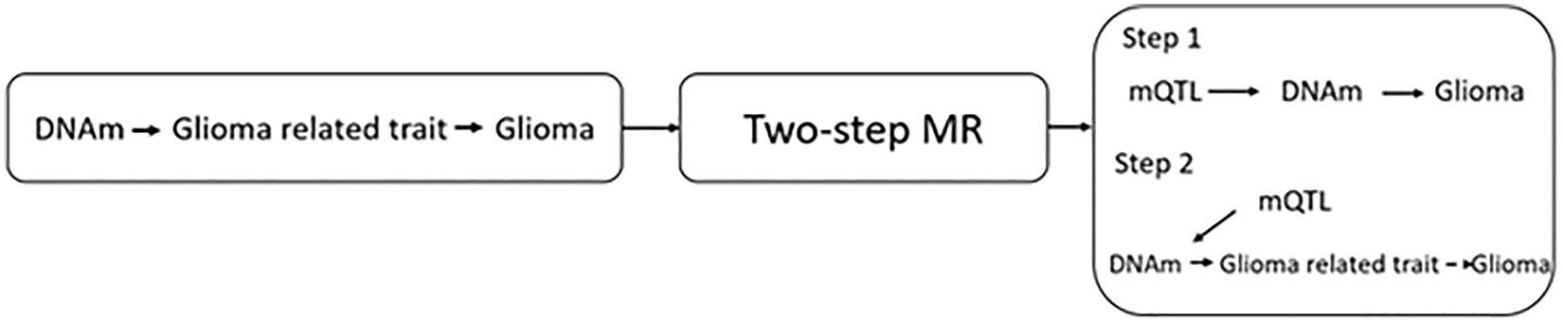
A summary of hypothesis 1. Does DNA methylation (DNAm) mediate the effect of the glioma related trait on glioma risk? MR, Mendelian randomization; mQTL, methylation quantitative trait loci.

#### Directionality test

To increase the likelihood that MR infers the correct causal direction between the exposure (e.g., DNAm) and the outcome (e.g., glioma), we applied the Steiger filtering method to test for reverse causation^60^. Steiger filtering removes SNPs that explain more of the variance in the outcome than the exposure and therefore the MR estimate is less likely to biased by misspecification in the MR model. Steiger filtering was performed for the putative causal variants identified in the MR analysis that showed evidence of colocalization.

### Hypothesis 1

A summary of the research questions addressed in hypothesis 1 is displayed in Fig. 6.

#### Step 1: evaluating the relationship between DNA methylation and glioma* risk*

##### Instrument selection

Two-sample MR was implemented to ascertain the potential causal effects of circulating DNAm on glioma risk. To create genetic IVs for DNAm as the exposure we used effect estimates for germline *cis*-SNPs (SNPs within a ± 250KBP window of the CpG site) robustly associated with DNAm at CpG site (mQTL) at genome wide significance (*P* < 5 × 10^–8^)^42^ that had undergone LD clumping (r^2^ < 0.001) from the mQTL database Genetics of DNA Methylation Consortium (GoDMC) [http://www.godmc.org.uk/] (n = 32,851)^42^. To measure instrument strength, we examined the variance in DNAm explained by the mQTLs (R^2^) and the F statistic^65^.

##### Outcome selection

For the glioma outcome, summary data were obtained from a GWAS meta-analysis of 12,488 glioma cases and 18,160 controls^66^. MR analyses were performed to assess the causal impact of DNAm variation on glioma subtypes: glioblastoma (6,183 cases) and non-glioblastoma (5,820 cases).

##### Mendelian randomization effect estimate and p-value threshold

MR effect estimates are reported as odds ratios (OR) (95% confidence intervals (CI)) per 1 standard deviation (SD) increase in genetically proxied DNAm.

#### Step 2: evaluating the relationship between DNA methylation and glioma related traits

##### Instrument selection

As described above, IVs for DNAm were generated (r^2^ < 0.001, *P* < 5 × 10^–8^) for CpG sites associated with either glioma, glioblastoma, and/or non-glioblastoma in step 1 above.

##### Outcome selection

For the outcome, summary data for the putative glioma related traits^24^ (genetically predicted leukocyte telomere length, allergic disease, alcohol consumption, childhood extreme obesity, LDLc and triglyceride levels) was obtained from MR-Base (a curated data base that contains complete GWAS results)^67^ (Table 9).

**Table 9:** The glioma related trait used an outcome in the MR analysis.

Glioma related trait	No of participants or no. cases	No. controls	Units	Pop	PubMed ID
Alcohol consumption	112,117	–	SD proxy	EUR	28937693
Allergic disease	180,129	180,709	Log odds	EUR	29083406
Low density lipoprotein cholesterol	441,016	–	SD	EUR	32203549
Obesity (early onset)	5530	8318	log odds	EUR	22484627
Telomere length	9190	–	SD	EUR	21573004
Triglycerides	441,016	–	SD	EUR	32203549

*SD* standard deviation, *Pop* population of the study participants.

##### Follow up tissue-specific Mendelian randomization* analysis.*

For the CpG sites that showed robust evidence of an effect with glioma risk, we investigated whether variation in tissue-specific gene expression was responsible for the effect with glioma risk. For the analysis we utilised blood tissue by incorporating gene expression data from the eQTLGen Consortium (n = 31,684) (https://www.eqtlgen.org/)^68^ and brain tissue utilising gene expression data from 13 brain tissues from The Genotype-Tissue Expression project (GTEx) v8 (n = 1194)^69^.

CpG sites were annotated to genes using the R package meffil^70^. IVs for genes were constructed using effect estimates for germline *cis*-SNPs (within a ± 250KBP window) associated with gene expression variation in brain and blood, namely expression quantitative trait loci (eQTLs) at genome wide significance (*P* < 5 × 10^–8^)^42^ that had undergone LD clumping (r^2^ < 0.001). To measure instrument strength, we examined the variance in gene expression explained by the eQTLs (R^2^) and the F statistic^65^.

##### Multiple trait colocalization

For genes that appeared to overlap with the CpG sites of interest we applied multiple trait colocalization (moloc)^71^ to investigate whether the same genetic variant influences proximal DNAm, proximal gene expression and glioma risk. Such analyses can provide evidence to support gene expression and DNAm residing on the same causal pathway to glioma onset^72^. We implemented “moloc” using data from three different data sources: DNAm data from the mQTL database GoDMC [http://www.godmc.org.uk/] (n = 32,851)^42^, gene expression data from the eQTLGen Consortium (n = 31,684) (https://www.eqtlgen.org/)^68^ and GWAS meta-analysis data for glioma^66^. Moloc default prior probabilities were implemented (p1 = 1 × 10^–4^, p2 = 1 × 10^–6^ and p3 = 1 × 10^–7^), p1 was used for one association, p2 for two associations, and p3 for colocalization of all three associations. We examined colocalization with expression of all genes with a ± 250KBP window of the CpG site of interest. At least 50 variants (minor allele frequency [MAF] > 0.05) common to all three datasets were required for the analysis. A posterior probability of greater than 70% was considered suggestive evidence of colocalization. All analyses were undertaken in R version 4.1.0.

### Hypothesis 2

**Figure 7:**
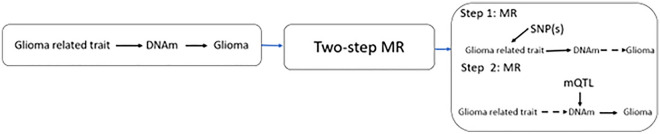
A summary of hypothesis 2: Does DNA methylation (DNAm) influence both glioma related traits and glioma risk? MR, Mendelian randomization; mQTL, methylation quantitative trait loci; SNP, single nucleotide polymorphism.

A summary of the research questions addressed in hypothesis 2 is displayed in Fig. 7.

#### Step 1: evaluating the relationship between glioma related trait and DNA methylation

Genetic instruments for the glioma related traits were collated from MR-Base^67^ or directly from the relevant GWAS (details of studies used to obtain genetic instruments are given in Table 10).

**Table 10:** A description of where summary effect estimates were sourced from to proxy the putative glioma related traits in the MR analysis.

Glioma related trait	No of participants or no. cases	No. controls	Pop	PubMed ID
Telomere length	9190	–	EUR	21573004
Allergic disease	180,129	180,709	EUR	29083406
Alcohol consumption	941,280	–	EUR	30,643,251
Obesity (early onset)	463,005		EUR	32376654
Low density lipoprotein cholesterol	441,016		EUR	32203549
Triglycerides	441,016	–	EUR	32203549

*Pop* population of study participants.

Genetic instruments were created using SNPs with an F statistic equal to or greater than 10, shown to be robustly (*P* < 5 × 10^− 8^) and independently (*r*^2^ < 0.001) associated with the glioma related trait under examination in individuals of European ancestry.

##### Outcome selection

For the outcome, summary data were obtained from the mQTL database GoDMC [http://www.godmc.org.uk/] (n = 32,851)^42^.

##### Mendelian randomization estimate and p-value threshold

The MR estimate was expressed as SD increase in methylation per unit increase in the glioma related trait. A Bonferroni-corrected *p*-value threshold, *P* value < 0.0083 (0.05/6 as there were 6 traits included in the analysis), was used to evaluate the strength of the statistical evidence.

#### Step 2: evaluating the relationship between DNA methylation associated with glioma related traits and glioma risk

Using IVs for the CpG sites that were influenced by putative glioma related traits, we examined if DNAm variation at these CpG sites had an MR effect on glioma risk using the glioma GWAS (12,488 glioma cases and 18,160 controls)^66^. MR effect estimates are reported as the OR (95% CI) per 1 SD increase in genetically proxied DNAm.

### Ethics approval and consent to participate

Ethical approval was not required for this specific analysis as the entirety of the data was sourced from the summary statistics of a published GWAS and no individual-level data were used.

## Supplementary Information

Supplementary Information.

## Data Availability

Genetic instrument for DNAm can be obtained from the mQTL database GoDMC [http://www.godmc.org.uk/] (n = 32,851). Genetic instruments used to proxy the six risk factors can be found through MR-Base (http://www.mrbase.org/) or from the individual reference papers. Meta-analysed glioma GWAS data were acquired from the study by Melin et al.^66^., which is a meta-analysis of eight independent GWAS studies (UK^73^, French^74^, German^75^, MDA^44^, UCSF-SFAGS^44^, GliomaScan^76^, GICC^64^ and UCSF/Mayo^77^). Genotype data from the Glioma International Case–Control Consortium Study GWAS are available from the database of Genotypes and Phenotypes (dbGaP) under accession phs001319.v1.p1. Genotypes from the GliomaScan GWAS can be accessed through dbGaP accession phs000652.v1.p1.

## Abbreviations

CI: Confidence interval
GWAS: Genome-wide association study
IV: Instrumental variable
MR: Mendelian randomization
IVW: Inverse variance weighted
OR: Odds ratio
SNP: Single nucleotide polymorphism
SD: Standard deviation
LDLc: Low-density lipoprotein cholesterol
DNAm: DNA methylation
EWAS: Epigenome-wide association study
mQTL: Methylation quantitative trait loci
CpG: Cytosine-phosphate-guanine
LD: Linkage disequilibrium
GoDMC: Genetics of DNA methylation consortium
